# The National Registry of pregnant women infected with HIV and of perinatally exposed children – a need for Romania?

**DOI:** 10.1186/1471-2334-14-S7-O8

**Published:** 2014-10-15

**Authors:** Mariana Mărdărescu, Cristina Petre, Ruxandra Drăghicenoiu, Rodica Ungurianu, Alina Cibea, Carina Matei, Sorin Petrea, Ana Maria Tudor, Delia Vlad, Marieta Iancu, Sanda Vintilă, Claudiu Mihai Șchiopu, Alexandra Mărdărescu, Ioana Alina Anca, Mihai Mitran

**Affiliations:** 1 National Institute for Infectious Diseases "Prof. Dr. Matei Balş", Bucharest, Romania; 2 Compartment for Monitoring and Evaluation of HIV Data in Romania, Bucharest, Romania; 3 The Institute for Mother and Child Protection “Alfred Rusescu”, Bucharest, Romania; 4 Clinical Hospital of Obstetrics and Gynecology “Prof. Dr. Panait Sârbu”, Bucharest, Romania

## Background

The recent years’ experience made us face a new typology of HIV cases (the outbreak within the people who inject drugs) as well as the necessity to adapt specific cares to the needs of the patients coming from the 1987-1990 cohort. The latter presents a complex pathology: they are therapeutically multi experienced, have adherence and ART resistance problems and experience advanced stages of disease. Furthermore, most of them have reached a fertile age, giving birth to a new generation of HIV children, which makes us update our approach to HIV infection. The National Registry of pregnant women- infected with HIV and of perinatally exposed children represents an operational tool that collects data on the item „mother-child”, whose main role is to clearly display a national overview on the phenomenon of mother to child transmission. The registry sides with a prospective observational study launched at 1 January 2014 that focuses on pregnant women and HIV exposed children from all the regions in Romania.

## Methods

The Registry stores personal data on both mothers and children, the child’s medical history (physiological and pathology data), initial investigations, investigations at 6 and 18 months of surveillance; the mother’s personal data, time of HIV diagnosis, risk factors, disease and therapeutic history, peripartum immunological and virological investigation, as well as information about the father and siblings.

## Results

Throughout 6 months of reporting we registered 97 cases of exposed children. 55% (54) come from newly detected mothers, 44% (43) from mothers belonging to the 1987-1990 cohort. 18% of children were also exposed to the drugs their mothers used, 18% to the mothers’ HCV and 26% to HBV. From the total number of children, 6.18% (6) had detectable viral load at birth. Regarding children infected with HIV, 4 were delivered naturally, 2 by C-section; 4 of them were breastfed and 2 received formula. All mothers in this group were late presenters, namely they were detected during pregnancy, during delivery or immediately after birth.

## Conclusion

Although HIV screening for pregnant women is free and universal in Romania, a high number of women are late presenters, which hinders any prophylactic measure to reduce the transmission of HIV from mother to child. Our 6 months assessment reveals that out of 97 exposed children, 6 presented detectable viral loads (6.18%) at the first screening. In all these cases the cause was the lack of or a precariously applied prophylaxis.

